# Triptolide-induced apoptosis in non-small cell lung cancer via a novel miR204-5p/Caveolin-1/Akt-mediated pathway

**DOI:** 10.18632/oncotarget.27672

**Published:** 2020-07-14

**Authors:** Brian J. Philips, Ajay Kumar, Sarah Burki, John P. Ryan, Kentaro Noda, Jonathan D’Cunha

**Affiliations:** ^1^Division of Lung Transplantation and Lung Failure, Department of Cardiothoracic Surgery, University of Pittsburgh, Pittsburgh, PA, USA; ^2^Department of Cardiothoracic Surgery, Mayo Clinic, Phoenix, AZ, USA

**Keywords:** triptolide, lung cancer, caveolin-1, miR204-5p, apoptosis

## Abstract

Lung cancer is one of the most prevalent malignancies world-wide with non-small cell lung cancer (NSCLC) comprising nearly 80% of all cases. Unfortunately, many lung cancer patients are diagnosed at advanced stages of the disease with an associated poor prognosis. Recently, the Chinese herb root extract Triptolide/Minnelide (TL) has shown significant promise as a therapeutic agent for NSCLC treatment both *in vitro* and *in vivo*. The aim of this study was to investigate the underlying mechanism(s) of action regarding TL-induced cytotoxicity in NSCLC. We demonstrate that triptolide treatment of A549 and H460 NSCLC cells decreases Caveolin-1 (CAV-1) mRNA/protein expression, resulting in activation of the Akt/Bcl-2-mediated mitochondrial apoptosis pathway. CAV-1 down-regulation was triggered by Micro-RNA 204-5p (miR204-5p) up-regulation and could be significantly blocked by pre-treatment with both Sirt-1/Sirt-3 specific siRNA and SIRT-1/SIRT-3 enzyme inhibitors, EX-527 and nicotinamide. Overall, our results provide evidence for a novel mechanism by which TL exerts its cytotoxic effects on NSCLC via CAV-1 down-regulation. Furthermore, these findings demonstrate a pivotal role for TL induction of the Akt/Bax pathway in apoptosis of human lung cancer.

## INTRODUCTION

Triptolide (TL), a diterpenoid triepoxide isolated from the Chinese plant *Tripterygium wilfordii* Hook F [1], is known to possess anti-inflammatory, immunosuppressive, and anti-tumor activities [2–4]. Numerous *in vitro* studies have revealed cytotoxic effects of TL in a wide range of human malignancies including breast [5, 6], pancreatic [7, 8], gastric [6, 9], colorectal [10, 11] and non-small cell lung carcinoma (NSCLC) [4, 12]. TL has also been reported to inhibit growth of solid tumors *in vivo* [4, 6]. Specifically, our group has shown that TL suppresses tumor growth in an established xenograft nude mouse model [4]. We recently provided evidence that TL decreases cell proliferation and induces apoptosis in NSCLC via inhibiting NF-kB signaling [4]. Moreover, our group has demonstrated that p53 deficiency exacerbates the cytotoxic effects of TL in NSCLC and that TL impairs mitochondria function in a p53-dependent manner by SIRT-3 regulation [12]. The SIRT proteins are a family of class III histone deacetylases that includes seven isoforms (SIRT1-7) in mammalian cells. These stress-responsive proteins, which have been implicated in carcinogenesis, are thought to have both tumor promoter and tumor suppressor functions depending upon the type of cancer [13, 14]. Furthermore, epigenetic regulation of Sirt mRNA, particularly Sirt-1 [15–18], by non-coding MicroRNAs (miRNAs) is believed to play a prominent role in cell proliferation, development and cancer formation [14]. miRNAs, a class of small non-coding RNAs involved in gene regulation, are known to be aberrantly expressed in human cancer cells (reviewed in [19]).

In recent years, considerable attention has focused on Caveolin-1 (CAV-1), a key scaffolding/signaling protein, in driving cancer progression and metastasis [20, 21]. As with many other cancers [22], overexpression of CAV-1 protein is associated with aggressiveness and metastasis, as well as poor clinical prognosis, in human lung cancer [23, 24]. Additionally, decreased expression of CAV-1 is associated with NSCLC drug-resistance [25, 26]. However, despite its known anti-proliferative/pro-apoptotic effects on NSCLC *in vitro*, the potential impact of TL on CAV-1 expression in lung cancer cells is unknown.

Because the precise mechanism(s) of the anti-lung cancer activities of TL are incompletely defined, we sought to determine if TL treatment of lung cancer cells affects both CAV-1 expression and overall cell viability. In this study, we found that TL induces Akt-mediated apoptosis in A549 and NCI-H460 NSCLC by direct down-regulation of CAV-1 mRNA/protein expression. We also demonstrate that TL significantly up-regulates miR-204-5p expression in both cell lines, resulting in decreased CAV-1 expression. These TL-mediated effects were significantly blocked by both siRNA and pharmacologic inhibition of SIRT-1 and SIRT-3 enzyme activity. Our *in vitro* findings suggest a novel mechanistic pathway by which TL triggers NSCLC cell death via CAV-1 down-regulation.

## RESULTS

### Triptolide downregulates CAV-1 and SIRT-1 mRNA/protein expression in human A549/NCI-H460 cells

**Figure 1:**
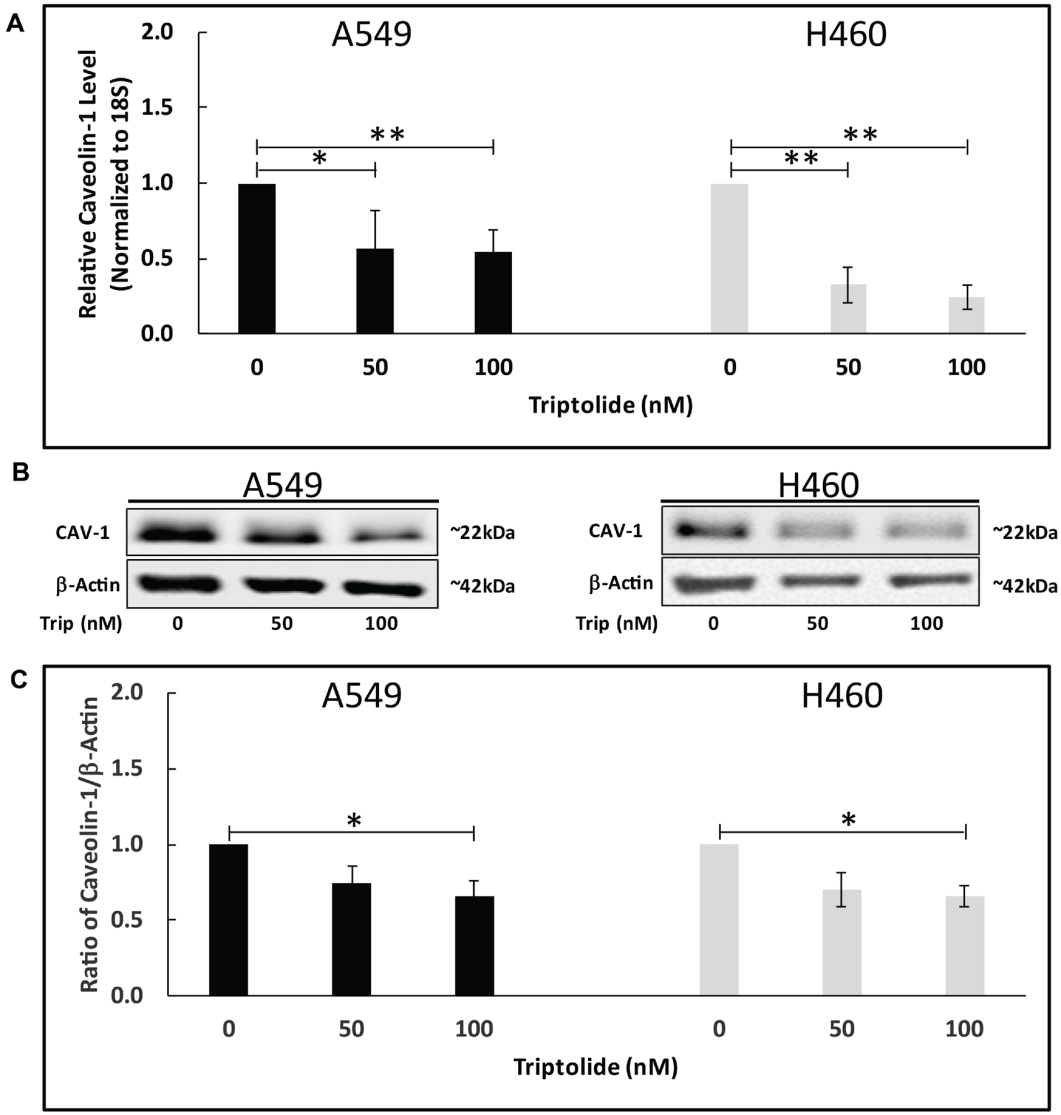
TL downregulated Cav-1 mRNA/protein expression in NSCLC. A549 and NCI-H460 cells were treated for 20 h ± 50 nM/100 nM TL. (**A**) Real-time RT-PCR was performed to analyze mRNA expression of *Cav-1* target gene. Values were normalized with *18S* rRNA expression and are expressed as mean ± SD. *n* = 5–7. ^*^indicates significantly different (*p* < 0.05). (**B**) Representative immunoblot and (**C**) quantitation of CAV-1 protein expression in A549 and NCI-H460 cells. Protein quantity was normalized to β-Actin. Data are presented as mean ± SD. *n* = 3–4. ^*^indicates significantly different (*p* < 0.05). ^**^indicates significantly different (*p* < 0.01).

Our earlier studies demonstrated that TL treatment of A549 and NCI-H460 NSCLC cells significantly decreased viability in a dose-dependent manner [4, 12]. Because CAV-1 over-expression is believed to be a key element in cancer development, we employed real-time reverse transcription PCR (RT-PCR) to explore whether TL affects Cav-1mRNA expression *in vitro*. Compared to control-treated cells, 50/100 nM TL treatment caused a significant reduction in mRNA expression in both A549 and H460 cells (Figure 1A). Specifically, in A549 cells, RT-PCR analysis showed an approximate 2-fold (45%) decrease (*p* < 0.05) in Cav-1 mRNA transcript levels with 50/100 nM TL, while decreased Cav-1 mRNA transcript levels in NCI-H460 cells were more pronounced with approximately 3-fold (65%) (*p* < 0.01) and 4-fold (75%) (*p* < 0.01) reduction with 50 nM and 100 nM TL treatment, respectively. In agreement with mRNA expression results, immunoblotting revealed a significant reduction (~30%, *p* < 0.05) in CAV-1 protein expression following 100 nM TL treatment of both A549 and NCI-H460 cells (Figure 1B and 1C).

**Figure 2:**
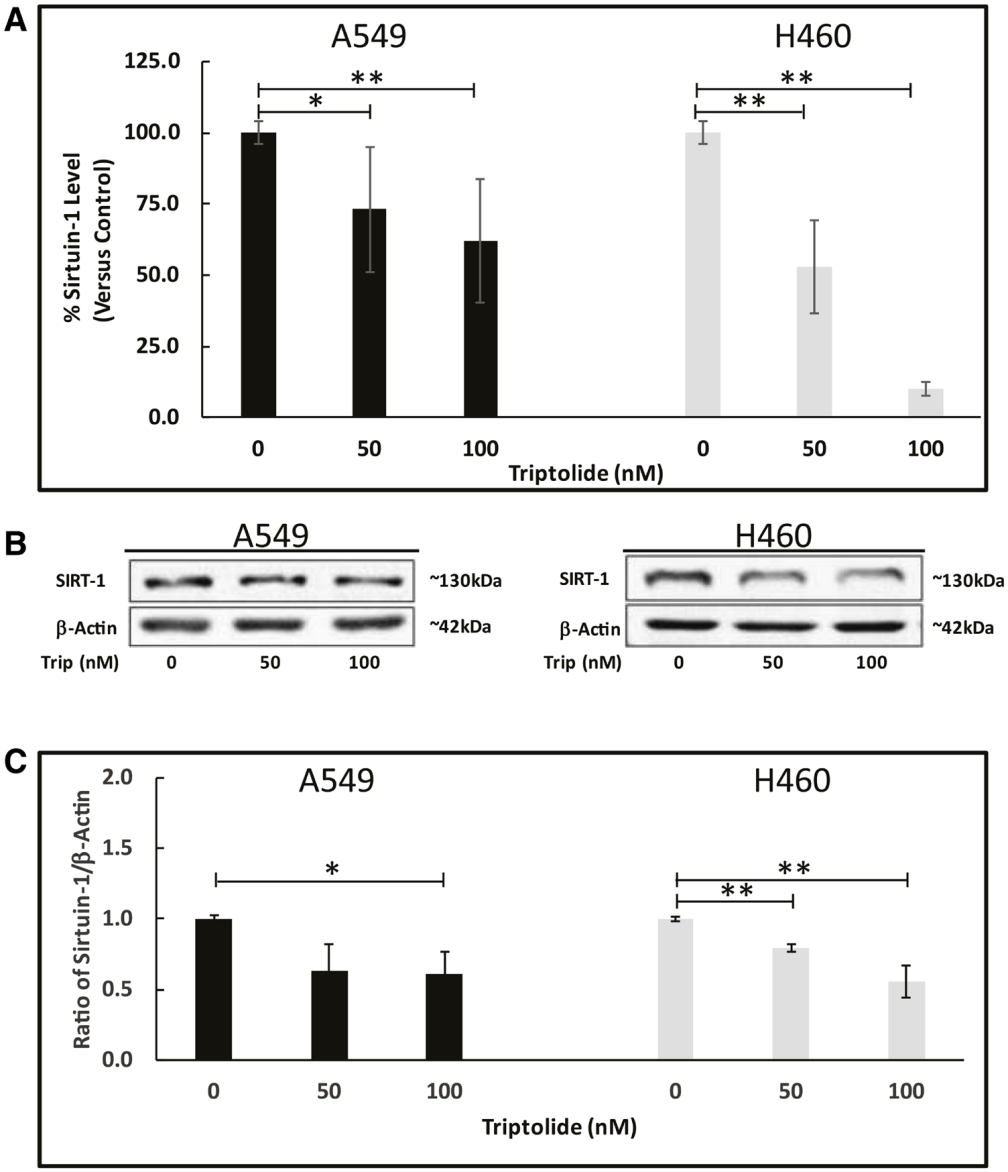
TL downregulated Sirt-1 mRNA/protein expression in NSCLC. A549 and NCI-H460 cells were treated for 20 h ± 50 nM/100 nM TL. (**A**) Real-time RT-PCR was performed to analyze mRNA expression of *Sirt-1* target gene. Values were normalized with *18S* rRNA expression and are expressed as mean ± SD. *n* = 6–8. ^*^indicates significantly different (*p* < 0.05). (**B**) Representative immunoblot and **(C**) quantitation of SIRT-1 protein expression in A549 and NCI-H460 cells. Protein quantity was normalized to β-Actin. Data are presented as mean ± SD. *n* = 3–4. ^*^indicates significantly different (*p* < 0.05). ^**^indicates significantly different (*p* < 0.01).

Next, we assessed mRNA/protein expression of SIRT-1, a major deacetylase believed to play a pro-tumorigenic role in lung cancer [25] and shown to be required for CAV-1 expression [17]. Similarly to CAV-1 expression results, we observed that TL treatment of A549 and NCI-H460 cells significantly (*p* < 0.05) reduced Sirt-1 mRNA and protein expression compared to non-treated cells. Sirt-1 mRNA transcript levels were reduced approximately 33% (*p* < 0.05) in A549 cells with 50/100 nM TL treatment, while NCI-H460 Sirt-1 mRNA levels decreased significantly in a dose-dependent manner with approximate decreases of nearly 2-fold (50%) (50 nM, *p* < 0.01) and 10-fold (90%) (100 nM, *p* < 0.01). (Figure 2A). Consistent with both CAV-1 protein and Sirt-1 mRNA expression results, immunoblot analysis indicated nearly 30% (*p* < 0.05) and 40% (*p* < 0.05) reduction, respectively, in SIRT-1 protein expression following 50 nM and 100 nM TL treatment of both A549 and NCI-H460 cells (Figure 2B and 2C). Altogether, these results indicate that TL significantly decreased both mRNA and protein expression of CAV-1 and SIRT-1 in NSCLC cells.

### SIRT-1 and SIRT-3 inhibition block triptolide-induced downregulation of CAV-1 expression in human A549/NCI-H460 cells

**Figure 3:**
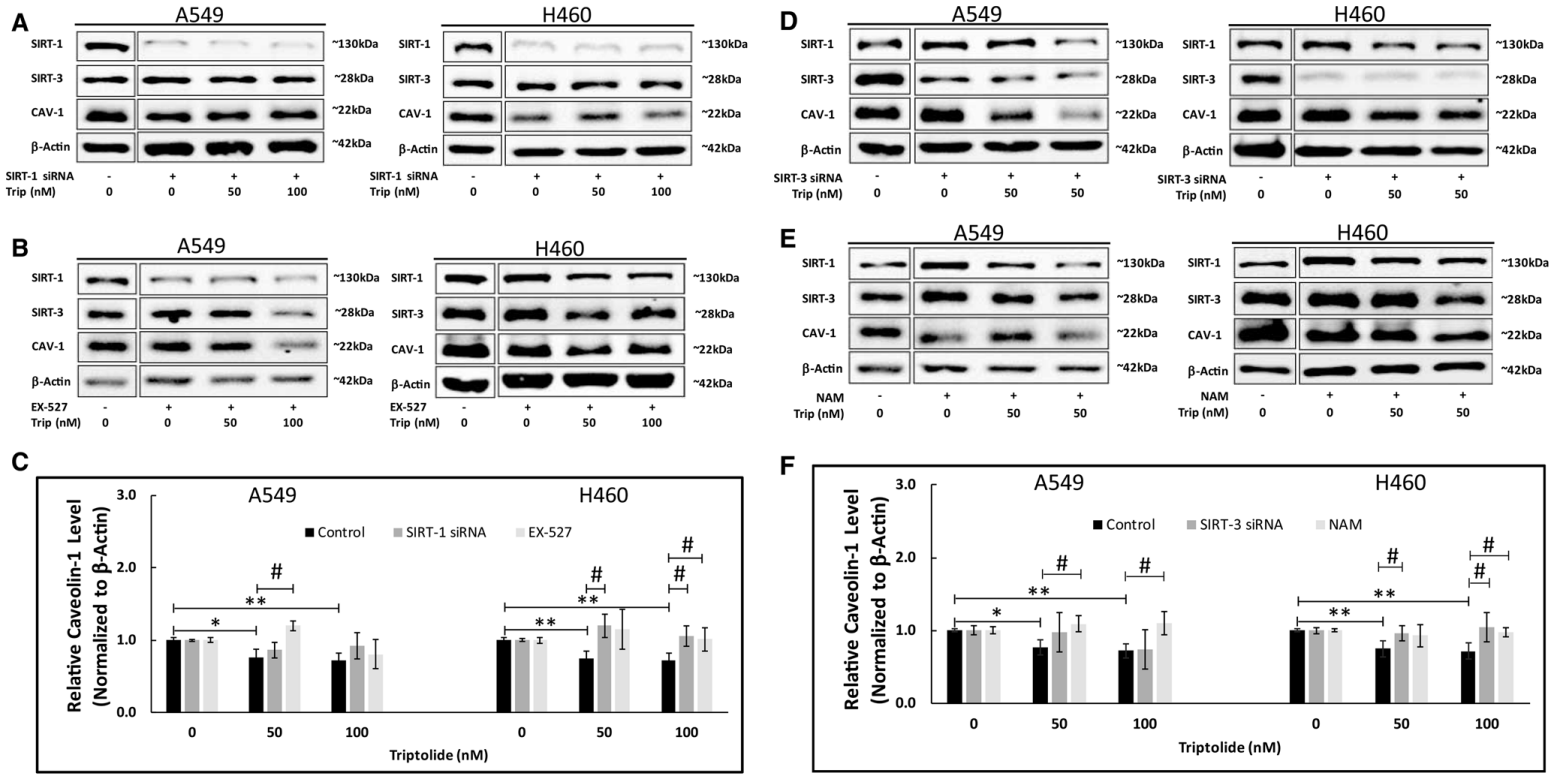
SIRT-1/-3 siRNA knockdown and EX-527/NAM treatment inhibited TL-induced downregulation of CAV-1 protein expression in NSCLC. A549 and NCI-H460 cells were treated for 4–6 h with Opti-MEM (low-serum) medium containing 80pmoles Sirt-1 siRNA (**A**), 10 μM EX-527 (**B**), 80pmoles Sirt-3 siRNA (**D**) or 10mM NAM (**E**). After 24 h (siRNA) or 4/6 h (EX-527/NAM), cells were incubated for 20 h ± 50 nM/100 nM TL. Representative immunoblots (A, B, D, E) and quantitation (**C**, **F**) for CAV-1 protein expression. Protein quantity was normalized to β-Actin. Data are presented as mean ± SD. *n* = 3–4. ^*^indicates significantly different (*p* < 0.05 vs non-TL treated). ^**^indicates significantly different (*p* < 0.01 vs non-TL treated). ^#^indicates significantly different (*p* < 0.05 vs control).

To further elucidate (and confirm) the apparent TL-induced mechanism for Sirt-1-mediated downregulation of Cav-1, we conducted two Sirt-1 mRNA inhibitory studies: 1) Pre-treatment with specific Sirt-1 siRNA and 2) Pharmacologic pre-treatment with EX-527, a selective Sirt-1 activity inhibitor [27, 28] and asked whether deficiency of Sirt-1 could prevent CAV-1 protein downregulation. As shown by immunoblot analysis in Figure 3A, specific siRNA-mediated downregulation of Sirt-1 mRNA impeded TL-induced CAV-1 protein down-regulation in both A549 (*p* > 0.05) and NCI-H460 cells (*p* < 0.05). In addition, pre-treatment with 10 μM EX-527 significantly (*p* < 0.05) blocked TL-induced CAV-1 protein down-regulation in both cell lines (Figure 3B and 3C). As expected, CAV-1 protein expression significantly (*p* < 0.05) decreased following TL treatment in control-treated (i.e., scrambled siRNA/No EX-527) cells (Figure 3C).

Our earlier study [12] supports an essential role of SIRT-3 for TL-induced impairment of mitochondrial function and associated apoptosis in A549 and NCI-H460 lung cancer cells. In a similar manner as SIRT-1 experiments above, we inhibited Sirt-3 mRNA expression via siRNA and Sirt-3 activity via nicotinamide chemical treatment [29] to determine whether SIRT-3 is involved in TL-mediated Cav-1 downregulation. Consistent with our previous findings [12], inhibition via Sirt-3 mRNA expression (Figure 3D and 3F; *p* < 0.05, NCI-H460) or SIRT-3 enzyme activity (Figure 3E and 3F; *p* < 0.05, A549/NCI-H460) significantly inhibited TL-induced CAV-1 protein down-regulation following TL treatment, indicating SIRT-3 appears necessary for TL/CAV-1 pathway in both NSCLC cells. Taken together, results from these inhibitor studies suggest SIRT-1 and SIRT-3 play important roles in mediating TL-induced down-regulation of CAV-1 protein expression in A549 and NCI-H460 lung cancer cells.

### Triptolide upregulates miR204-5p expression, causing downregulation of Cav-1 and Sirt-1 mRNA expression in human A549/NCI-H460 cells

**Figure 4:**
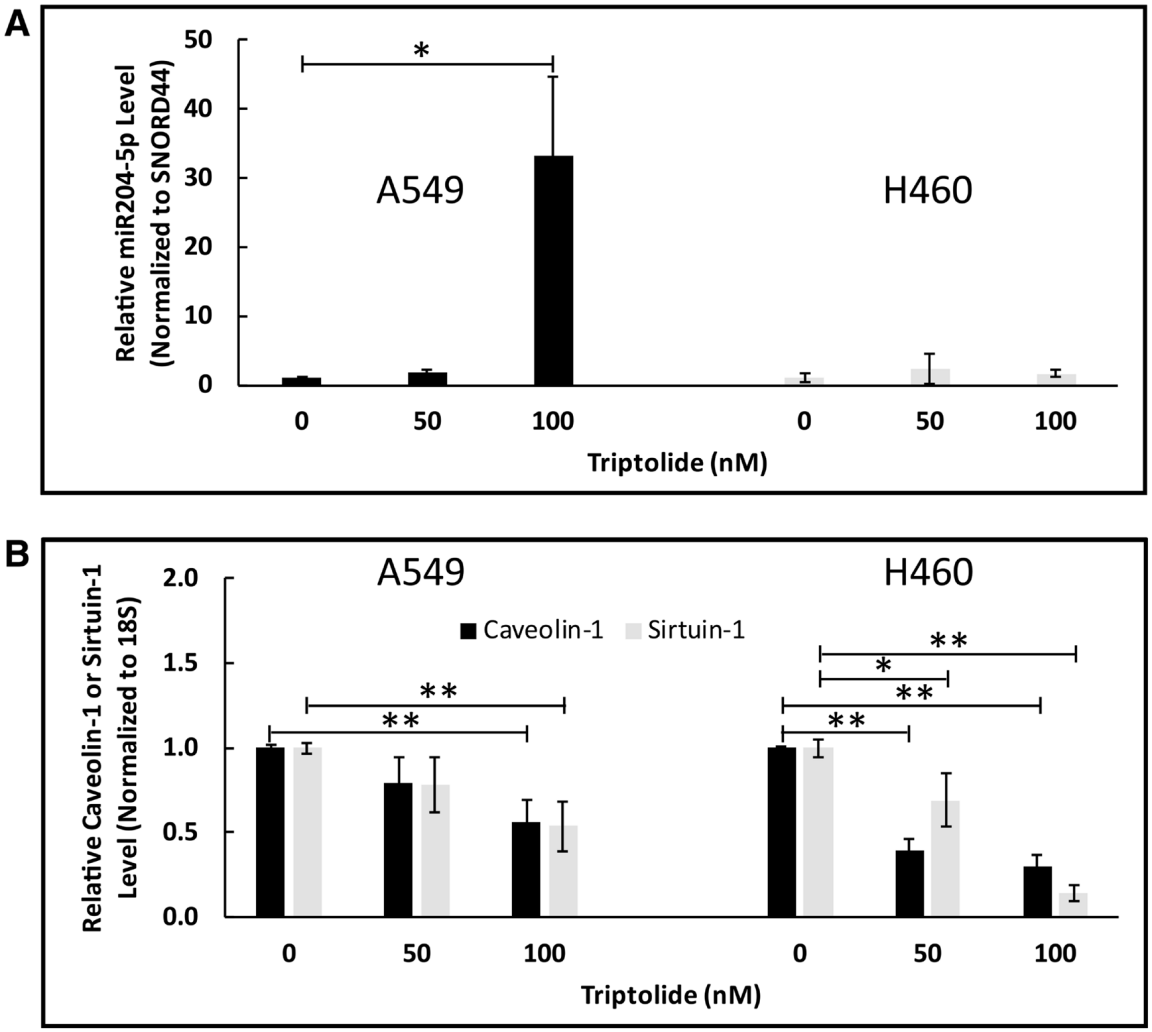
TL upregulated miR204-5p mRNA expression in NSCLC. A549 and NCI-H460 cells were treated for 20 h ± 50 nM/100 nM TL. Real-time RT-PCR was performed to analyze mRNA expression of (**A**) *miR204-5p* and (**B**) *Cav-1/Sirt-1* target genes. Values were normalized with either (A) *Snord44* or (B) *18S* rRNA expression and are expressed as mean ± SD. *n* = 3–4. ^*^indicates significantly different (*p* < 0.05). ^**^indicates significantly different (*p* < 0.01).

**Figure 5:**
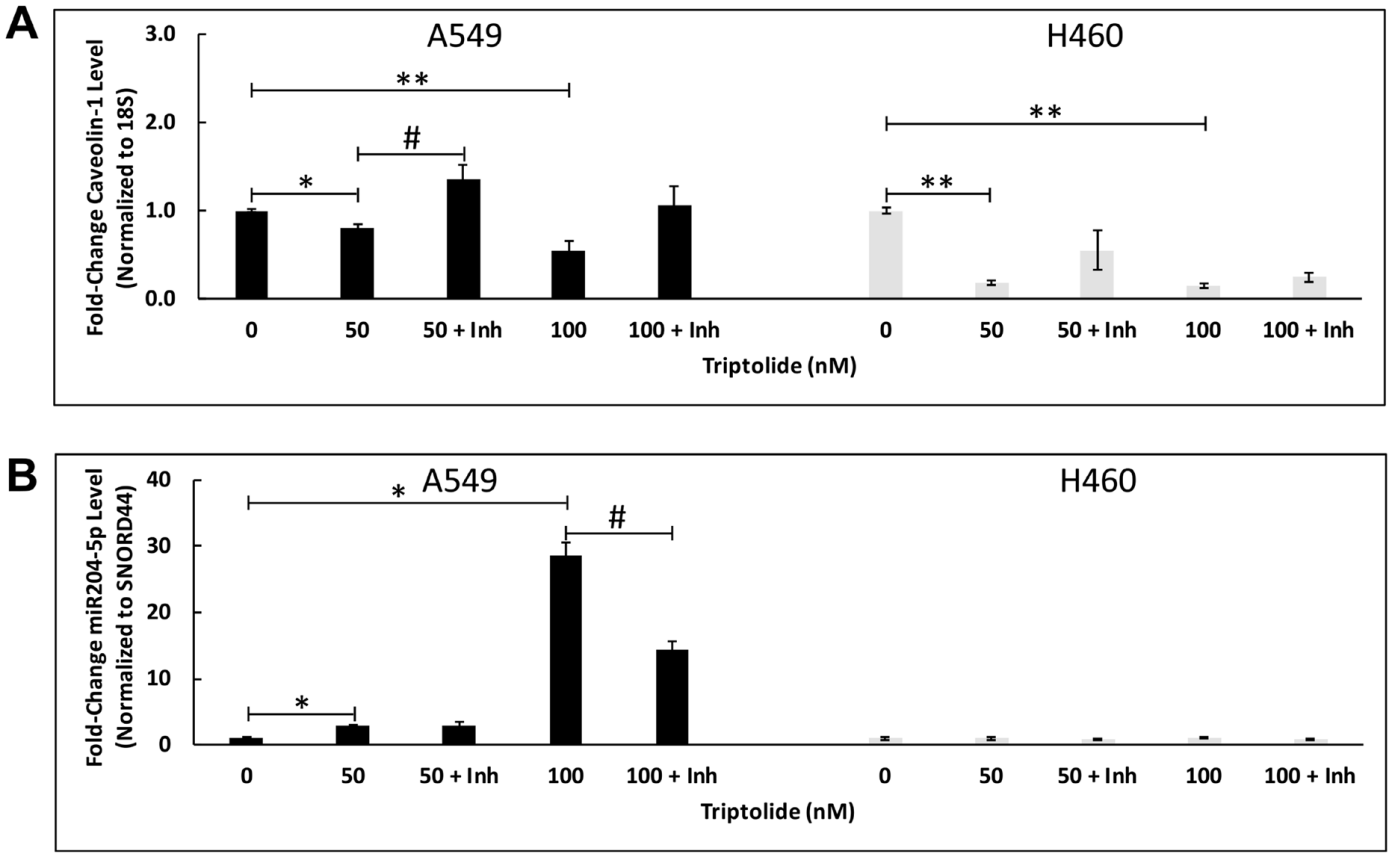
miR204-5p inhibitor blocked TL-induced downregulation of Cav-1 mRNA expression in NSCLC. A549 and NCI-H460 cells were first treated for ~24 h with Opti-MEM (low-serum) medium containing miR204-5p inhibitor or scrambled control miRNA, then incubated for 20 h ± 50 nM/100 nM triptolide. Real-time RT-PCR was performed to analyze mRNA expression of (**A**) *Cav-1* and (**B**) *miR204-5p* target genes. Values were normalized with either (A) *18S* rRNA or (B) *Snord44* expression and expressed as mean ± SD. *n* = 3. ^*^ indicates significantly different (*p* < 0.05 vs non-treated). ^**^indicates significantly different (*p* < 0.01 vs non-TL treated). ^#^indicates significantly different (*p* < 0.05 vs TL alone).

Over the last decade, non-coding MicroRNAs (miRNAs) have garnered considerable attention for their potential use as a tool for cancer diagnosis and prognosis. Indeed, previous studies in human malignancies have shown Sirt-1 to be a target gene of miR204-5p [25–27]. To explore the potential effect of TL on miR204-5p expression in NSCLC, we examined miR204-5p transcript levels in A549 and NCI-H460 cells using RT-PCR. As shown in Figure 4A, we found that TL treatment significantly (100 nM, *p* < 0.05) upregulated miR204-5p RNA expression in a dose-dependent manner (2.5-30-fold) in A549 cells versus non-treated cells. In H460 cells, miR204-5p RNA expression was elevated modestly (~1.7-fold, *p* > 0.05) compared to control-treated cells following 50/100 nM TL treatment. As expected, mRNA expression of both Cav-1 and Sirt-1 significantly (*p* < 0.05) decreased following treatment with TL (Figure 4B). To further validate these findings, A549 and NCI-H460 cells transfected with miR204-5p inhibitor (prior to TL treatment) demonstrated increased (*p* < 0.05, A549) Cav-1 (Figure 5A) mRNA transcript expression, along with decreased (*p* < 0.05, A549) mRNA expression of miR204-5p (Figure 5B), in comparison to control (scrambled siRNA) treated cells. No significant differences in mRNA levels of Cav-1 and miR204-5p were exhibited with control (scrambled siRNA) treated cells ± TL versus TL alone (non-siRNA)-treated cells (data not shown). Overall, these findings suggest that miR204-5p plays a role in TL-induced down-regulation of Cav-1/Sirt-1/-3 in A549 and NCI-H460 NSCLC cells.

### TL induces Akt/Bax-dependent apoptosis in human A549/NCI-H460 cells

**Figure 6:**
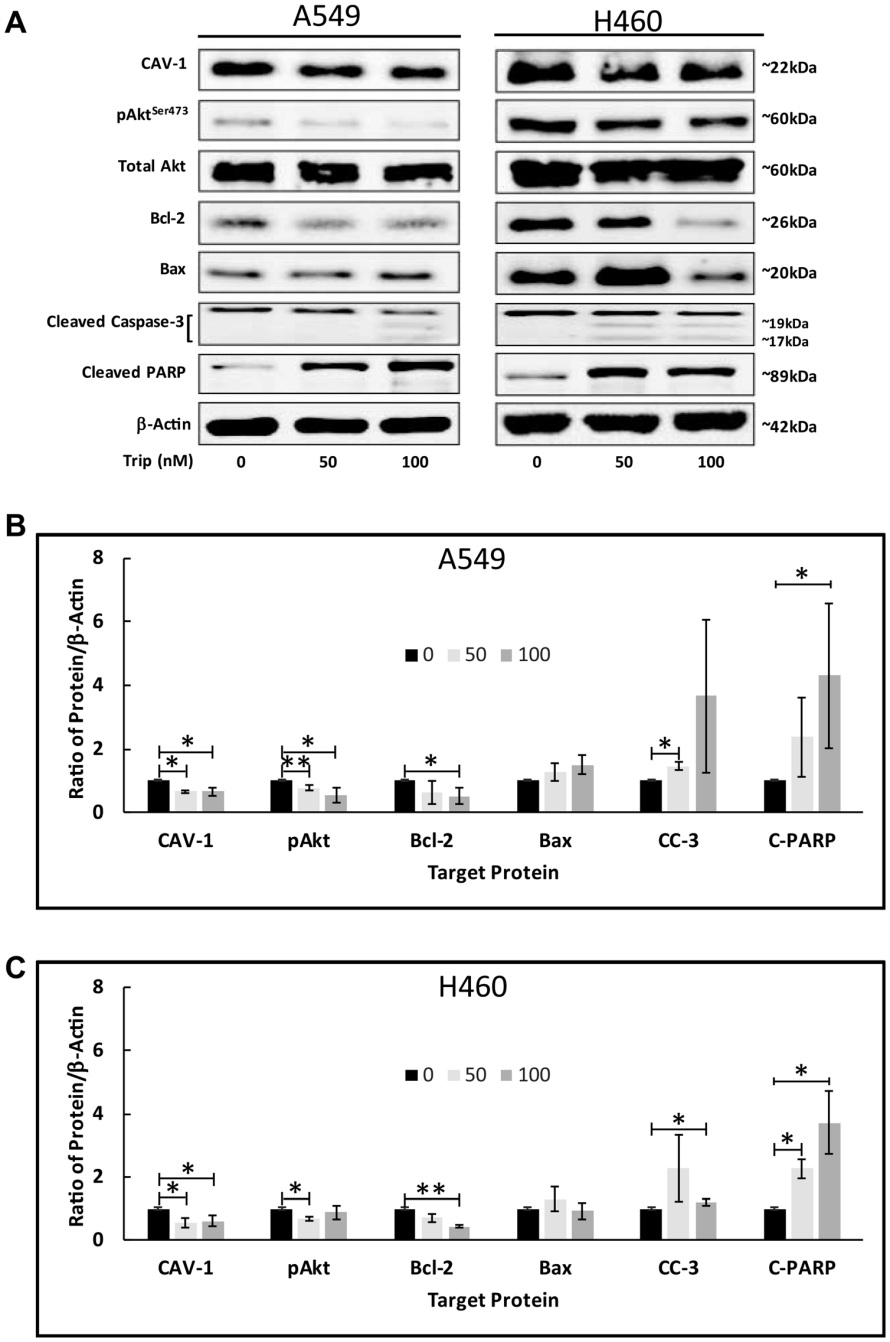
TL induced Akt activation in NSCLC. A549 and NCI-H460 cells were treated for 18–24 h ± 50 nM/100 nM TL. Representative immunoblots (**A**) and quantitation (**B**, **C**) for CAV-1, pAkt (Ser473), Total Akt, Bcl-2, Bax, Cleaved caspase-3 (CC-3) and Cleaved-PARP (C-PARP) protein expression. Protein quantity was normalized to β-Actin. Data are presented as mean ± SD. *n* = 3–4. ^*^ indicates significantly different (*p* < 0.05). ^**^ indicates significantly different (*p* < 0.01).

Previously, we have demonstrated that: 1) TL promotes apoptosis in various NSCLC by regulating key apoptotic factors [4] and 2) TL impairs mitochondria function in a p53-dependent manner by SIRT-3 regulation [12]. To investigate the underlying mechanisms of TL/CAV-1-induced apoptosis, we analyzed protein expression of key markers associated with the mitochondrial apoptosis pathway following treatment with 50/100nM TL. As shown in Figure 6A, both A549 and NCI-H460 cells expressed considerable levels of endogenous phosphorylated Akt (Ser473), suggesting Akt activation in these cells. Following TL treatment, we observed a significant (*p* < 0.05) attenuation of Akt phosphorylation, along with significantly (*p* < 0.05) decreased CAV-1 protein expression in both cell lines (Figure 6A and 6B). Examination of further downstream targets indicated TL treatment significantly (*p* < 0.05) increased the (pro-apoptotic/anti-apoptotic) Bax/Bcl-2 ratio and activated the mitochondrial apoptotic pathway, inducing cleaved caspase-3 and cleaved-PARP (Figure 6A–6C). Collectively, these results suggest that apoptotic effects of TL in A549 and NCI-H460 NSCLC cells are mediated via the activation of the classical Akt/Bax mitochondrial signaling pathway.

## DISCUSSION

Triptolide (TL) has shown considerable promise as an effective anti-proliferative/-neoplastic agent both *in vitro* and *in vivo*. Clinically, such encouraging effects are currently being evaluated in both national and international, multi-center phase I and II trials (clinicaltrials.gov) using Minnelide which is the water-soluble prodrug developed for clinical use. Previously, we have shown that TL suppresses solid tumor growth in mice via apoptotic induction [4]. However, because its molecular mechanism(s) of action are not completely understood, we sought to pursue a deeper understanding of the basic molecular interactions responsible for TL’s anti-growth/pro-apoptotic effects *in vitro*. In the present study, we explored the potential involvement of CAV-1, a key signaling protein associated with cancer progression, regarding TL-induced cytotoxicity in A549 and NCI-H460 lung cancer cells. Our results indicate that TL significantly decreases CAV-1 mRNA/protein expression, causing apoptosis in A549 and NCI-H460 cells by activation of the classical Akt/Bcl-2-mediated mitochondrial apoptosis pathway. In addition, our data demonstrate that CAV-1 down-regulation, coupled with cellular apoptosis, could be significantly blocked by inhibition of miR204-5p, SIRT-1 and SIRT-3.

CAV-1 is an integral membrane protein involved in cell signaling and transport. For many human tumors, CAV-1 upregulation influences cancer cell survival and growth, thus favoring tumor progression [30]. In lung cancer, overexpression of CAV-1 protein is known to be associated with aggressiveness and metastasis, as well as poor clinical prognosis [23, 24]. In addition, Han *et al.* [31] showed that CAV-1 knockdown caused a significant reduction in cell growth of paclitaxel-resistant lung cancer A549 cells. More recently, studies by Liu and coworkers [32] demonstrated that knockdown of CAV-1 in A549 cells enhanced cisplatin-triggered cancer death. Because of the growth inhibition and associated apoptotic effects of TL in a wide variety of epithelial and hematological cancer cell lines [5–11], including NSCLC cells [4, 12], we investigated the potential changes in CAV-1 mRNA/protein expression following TL treatment in A549 and NCI-H460 lung cancer cells.

Our *in vitro* data showed that 50/100 nM TL treatment of A549 and NCI-H460 cells caused a significant (*p* < 0.05) reduction in both CAV-1 mRNA (Figure 1A) and protein (Figure 1B and 1C) expression in both A549 and NCI-H460 cells. Specifically, decreased Cav-1 mRNA transcript levels in NCI-H460 cells were more pronounced (~3-4-fold) than in A549 cells (~2-fold), though effects on decreased protein expression were similar for both cell lines. In agreement with Han *et al.* [31] and Liu *et al.* [32] findings, these observations suggest that CAV-1 down-regulation plays an important role regarding TL-induced cytotoxicity in A549 and NCI-H460 NSCLC cells.

Sirtuins are members of the class III histone deacetylase family of enzymes that influence the cellular responses to genomic instability by regulating the cell cycle, DNA repair, cell survival and apoptosis, and thus have pivotal roles in cancer initiation and progression [33]. In 2001, SIRT-1 was the first sirtuin shown to be involved in cancer via repression of p53 activity [34, 35]. Moreover, it has recently been demonstrated that SIRT-1 is required for Cav-1 expression [17] and that CAV-1 is a direct binding partner of SIRT-1 under oxidative stress conditions [36]. As with CAV-1 mRNA/protein expression, we found that TL treatment of A549 and NCI-H460 cells significantly (*p* < 0.05) reduced Sirt-1 mRNA and protein expression versus control-treated cells (Figure 2), consistent with previous findings [17]. Compared to A549 cells, NCI-H460 Sirt-1 mRNA transcript expression was particularly sensitive to TL as evidenced by the significant (*p* < 0.01) dose-dependent decrease in expression (Figure 2A), though decreased protein expression profiles (~35% reduction) were comparable for both cell lines (Figure 2B and 2C). These data strongly imply that SIRT-1 is necessary for CAV-1 expression and implicated a potential link between SIRT-1/CAV-1 expression in mediating the effects of TL.

Our previously published findings [12] established an essential role of SIRT-3 for TL-induced impairment of mitochondrial function and associated apoptosis in A549 and NCI-H460 lung cancer cells. To further substantiate the roles of SIRT-1 and SIRT-3 involved in TL-triggered CAV-1 repression, we treated both cell lines with SIRT-1/-3 siRNA and with the enzyme inhibitors EX-527/nicotinamide. As shown by Western blot (Figure 3A and 3D) and protein quantitation (Figure 3C and 3F) analyses, specific siRNA-mediated knockdown of either Sirt-1 or Sirt-3 effectively blocked TL-induced CAV-1 protein down-regulation in both A549 and NCI-H460 cells. Quantitation of CAV-1 protein expression indicated that siRNA knockdown of Sirt-1/-3 mRNA generated significant (*p* < 0.05) effects only in NCI-H460 cells (Figure 3C and 3F). It is plausible that reduced transfection efficiency, decreased siRNA sensitivity and/or insufficient cell exposure time to Sirt-1/-3 siRNA prior to TL treatment may explain these abridged CAV-1 expression effects in A549 cells. Treatment of A549 cells with increased (> 80 nM) Sirt-1/-3 siRNA may significantly reduce CAV-1 expression, although based upon SIRT-1/-3 endogenous (non-treated) protein (Figures 2 and 3) and mRNA levels (data not shown), we observed that A549 and NCI-H460 cells have very similar expression profiles.

Additionally, opposing effects of TL and SIRT-3 knockdown may be responsible for the disparate findings in A549 cells. Specifically, Sirt-3 mRNA knockdown suppressed CAV-1 protein expression downregulation, but TL treatment concomitantly decreased Sirt-1 mRNA/protein expression (Figure 2) resulting in decreased CAV-1 protein expression. Consequently, it would be expected that under these conditions, Sirt-3 mRNA knockdown effects would be muted regarding suppression of CAV-1 protein expression. Such effects were not observed in NCI-H460 cells under Sirt-3 mRNA knockdown conditions perhaps due to uncharacterized inherent cell line differences, though future studies with double-knockdown Sirt-1/-3 using low (< 25 nM) doses of TL would help elucidate the mechanistic contributions of each SIRT protein. Such low TL doses have been reported to have minimal effects on A549 growth and viability after 24 h treatment [4, 37].

Furthermore, pre-treatment of both cell lines with either 10 μM EX-527 or 10 mM NAM significantly (*p* < 0.05) inhibited TL-induced CAV-1 protein down-regulation (Figure 3C and 3F), suggesting that (under our conditions) SIRT enzyme inhibition was a more effective means than siRNA pre-treatment to block CAV-1 protein down-regulation. Nonetheless, these inhibitor studies indicate that SIRT-1 and SIRT-3 play pivotal roles in mediating TL-induced down-regulation of CAV-1 protein expression in A549 and NCI-H460 lung cancer cells, further supporting the importance of SIRT-3 in TL-mediated cytotoxicity [12].

Because of its wide-spread apoptotic effects in cancer cells, we were interested in screening TL-treated lung cancer cells for cDNA profile expression changes. Microarray studies (data not published) demonstrated that following treatment of A549 cells with 50 nM and 100 nM TL for 20 h, one of the most highly-expressed gene transcripts identified was miR204-5p. MicroRNAs (miRNAs) are a family of small non-coding RNAs that regulate numerous biological processes, and their expression has been found to be highly dysregulated in cancer cells (reviewed in [19]). Regarding lung cancer, Hu *et al.* [38] identified four specific miRNA signatures that were significantly associated with overall survival of lung cancer. Also, several more recent *in vitro* studies have revealed a significant reduction in NSCLC cell proliferation with specific miRNA overexpression [39, 40].

Our *in vitro* studies indicated that 50/100 nM TL treatment of A549 cells caused significantly increased miR204-5p RNA expression in a dose-dependent manner (*p* < 0.01 at 100 nM) versus non-treated cells (Figure 4A), whereas miR204-5p RNA expression was elevated modestly (~2-fold, *p* > 0.05) in NCI-H460 cells compared to DMSO-treated cells. More importantly, mRNA expression of both Cav-1 and Sirt-1 significantly (*p* < 0.05) decreased concomitantly with miR204-5p upregulation (Figure 4B) implying that elevated miR204-5p RNA is associated with TL-induced cytotoxicity in A549 and NCI-H460 lung cancer cells. One potential reason for the discrepancy in TL-induced miR204-5p expression between the two cell lines could be the endogenous transcript levels for each cell line. Relative to basal 18S RNA expression, our RT-PCR studies indicated that H460 cells possess nearly 500- to 1000-fold increased basal expression of miR204-5p than A549 cells (data not shown). As a result, any induction of miR204-5p RNA by TL treatment would be more pronounced in A549 cells compared to NCI-H460 cells. Notably, treatment of H460 cells with a higher dose (200 nM) of TL did not increase miR204-5p transcript expression above the level observed at 100 nM TL (data not shown).

To further confirm and validate a link between miR204-5p expression and CAV-1 expression, we demonstrated that transfection of A549 and NCI-H460 cells with a specific miR204-5p inhibitor (prior to TL treatment) effectively repressed the TL-induced inhibitory effect on Cav-1 mRNA transcript expression (Figure 5A). These findings are consistent with reports of Cav-1 being a target of miR204-5p [17, 41–44]. More specifically, our data substantiate very recent findings by Huang *et al.* [44] that downregulation of miR-204 expression is responsible for CAV-1 overexpression in cisplatin-resistant A549 cells. Taken together, our findings indicate that: 1) Cav-1 is a likely target of miR204-5p and 2) miR204-5p plays a key role in TL-induced down-regulation of Cav-1/Sirt-1/-3 in A549 and NCI-H460 NSCLC cells.

In attempt to further investigate the precise molecular mechanism of TL-induced cytotoxicity, protein expression of several downstream signaling molecules was analyzed by immunoblotting following 18–24 h TL treatment of A549 and NCI-H460 cells. We found that TL-mediated Cav-1 knockdown significantly (*p* < 0.05) reduced the phosphorylation level of Akt (Ser473) in both cell lines (Figure 6). Additionally, Cav-1 repression by TL treatment significantly (*p* < 0.05) increased the (pro-apoptotic/anti-apoptotic) Bax/Bcl-2 ratio and activated the canonical mitochondrial apoptotic pathway, inducing the Caspase-3 cascade effect and the expression of cleaved PARP (*p* < 0.05, Figure 6). These observations are consistent with our previous study [4], which showed both significantly increased Caspase-3 enzyme activity and cleaved-PARP production after 24 h 100/200 nM TL treatment of A549 and NCI-H460 cells. Also, our current data contradict previous reports [45] of unobserved apoptosis (despite ~50% cell death) in A549 cells treated for 24 h with 200 nM tripchlorolide, a derivative of TL. Moreover, our findings are in agreement with previous studies that have demonstrated activation of the Akt signaling pathway/apoptosis with similar time/TL dosage treatment of cancer cells [7], including A549 cells [46–49]. Overall, our study indicated that TL promoted apoptosis in NSCLC cells through Akt pathway signaling.

Our study is not without its limitations. For example, numerous studies have firmly demonstrated that Cav-1 is a direct target of miR204-5p [17, 41–44], thus miR-204-5p could directly down-regulate protein expression of CAV-1 in NSCLC. To our knowledge, this direct connection has not been demonstrated in NSCLC though based upon our findings, CAV-1 over-expression studies using SIRT-1 or SIRT-3 knockout cells would be a viable means to test this hypothesis. Also, double-knockdown SIRT-1/-3 using low (< 25 nM) doses of TL would help elucidate the mechanistic contributions of each SIRT protein. Furthermore, several studies have reported that in human cancers, including NSCLC, the JAK1/STAT3 pathway (which was not examined here) is blocked/inhibited upon TL treatment. Thus, this pathway is a possibility of future exploration for drug development in the treatment of NSCLC. Lastly, our group has previously shown that TL suppresses tumor growth in an established xenograft nude mouse model. Analysis by RNA-Seq on these tumor tissues would provide valuable information regarding additional TL-regulated genes. It should be noted that our standard approach is to perform the experiments Reviewers suggest for improvement and clarity of our work; however, due to the COVID-19 pandemic, research facilities at the University of Pittsburgh are limited in access for the unforeseeable future. Consequently, any additional experiments for this study are not feasible at this time.

**Figure 7:**
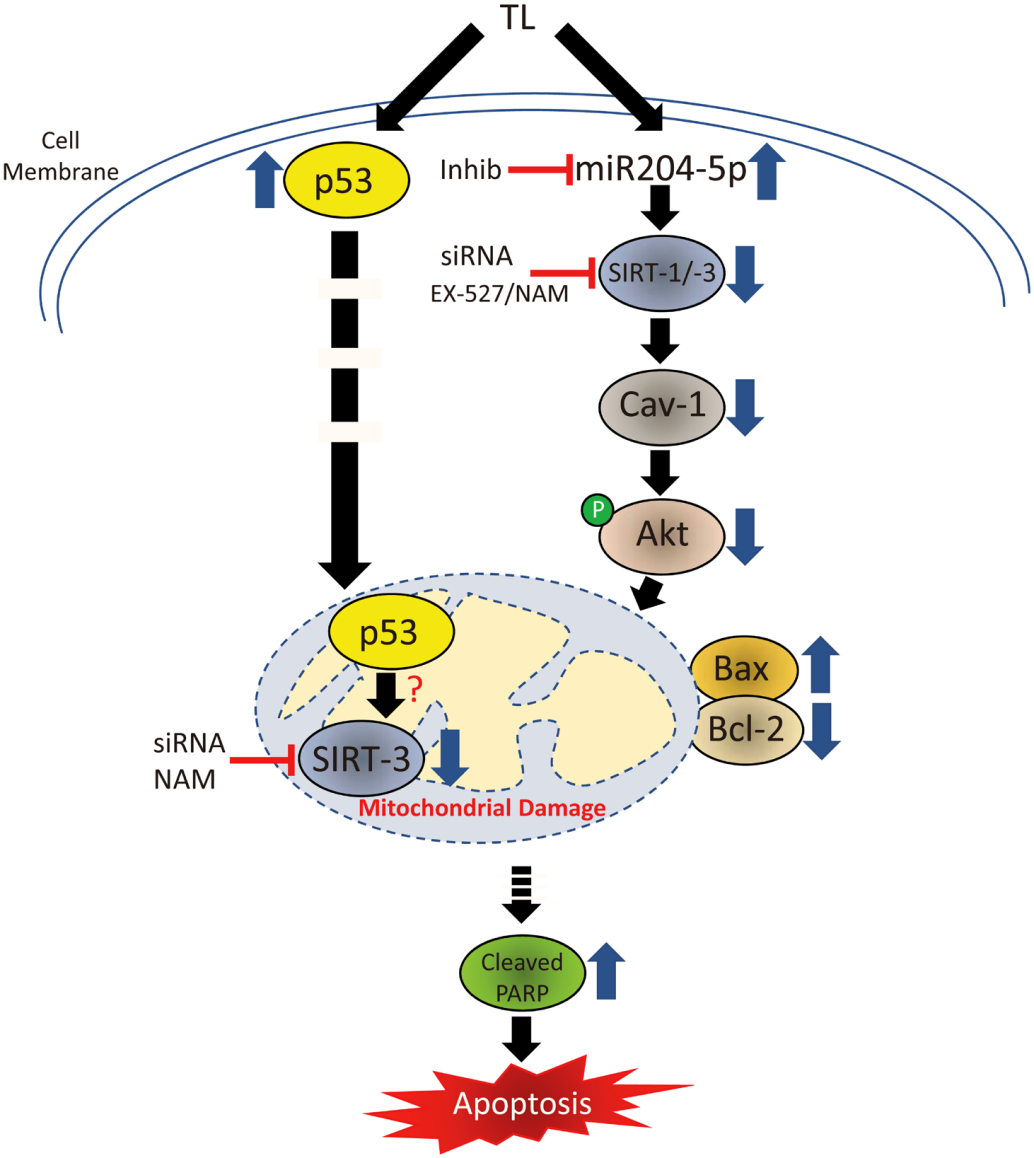
Signal transduction cascade mediating TL-induced apoptosis in NSCLC. TL decreases protein expression of both SIRT-1 and SIRT-3 through independent pathways resulting in mitochondrial dysfunction in NSCLC.

In summary, our results indicate that: 1). Inhibition of Sirt-1 or Sirt-3 via siRNA or pharmacological inhibition and 2). Inhibition of miR-204-5p via siRNA are able to effectively block TL-induced CAV-1 protein down-regulation in both A549 and NCI-H460 NSCLC cell lines. These findings strongly suggest a link between miR-204-5p and SIRT-1:-3/CAV-1 expression in mediating the apoptotic effects of TL in NSCLC and indicate a novel mechanism by which TL, via miR204-5p up-regulation and associated CAV-1 down-regulation, triggers the activation of mitochondria-mediated apoptosis in NSCLC (Figure 7). Mechanistically, TL decreases phosphorylation of Akt (Ser473), causing Bcl-2 down-regulation and Bax up-regulation, culminating in caspase-3 activation and cleavage of PARP. In this scenario, nuclear full-length (FL) SIRT-3, following TL treatment of NSCLC (i.e., cellular stress), likely mediates Cav-1 mRNA down-regulation either directly or indirectly. Overall, this study proposes new insight into the mechanistic basis of TL-induced mitochondria-mediated apoptosis involving miR204-5p/CAV-1 and provides a rationale for future clinical investigation of the therapeutic efficacy of TL in NSCLC patients.

## MATERIALS AND METHODS

### Cell culture conditions

The human NSCLC cell lines A549 (K-Ras mutant, adenocarcinoma) and NCI-H460 (K-Ras mutant, adenocarcinoma) were obtained from the American Type Culture Collection (Rockville, MD, USA). Cells were cultured in a humidified incubator at 37°C and 5% CO2 and grown in RPMI 1640 medium (Life Technologies Inc, Carlsbad, CA, USA) supplemented with 10% heat-inactivated fetal bovine serum, 50 U/mL penicillin and 50 μg/mL streptomycin (Life Technologies Inc). Cell media was replaced every 48 h and cells were split upon 80–90% confluence.

### Reagents and antibodies

Triptolide (TL), purchased from Calbiochem (NJ, USA), was dissolved in Dimethyl sulfoxide (DMSO, Sigma-Aldrich, St. Louis, MO, USA) to a stock solution of 1 mg/ml (2.77 mM) and stored at –20°C. Nicotinamide (NAM), obtained from Sigma-Aldrich, was prepared fresh prior to each experiment by dissolving in 1× phosphate buffered saline (PBS) to a stock solution of 122 mg/mL (1M). EX-527 (Sigma-Aldrich) was dissolved in 100% DMSO to a stock solution of 20 mM and stored at –20°C. Cells were stimulated with indicated doses of TL, NAM or EX-527 in complete media. DMSO alone was included to serve as a control. All data were acquired from at least 3 independent experiments.

The following primary antibodies were utilized for immunoblotting: rabbit polyclonal anti-caveolin-1 (N-20, 1:1000, Santa Cruz Biotechnology, Santa Cruz, CA, USA), mouse monoclonal anti β-actin (C4, 1:10,000, Santa Cruz), rabbit monoclonal anti-SIRT-1 (D1D7, 1:1000, Cell Signaling Technology, Danvers, MA, USA), rabbit monoclonal anti-SIRT-3 (C73E3, 1:1000, Cell Signaling), rabbit polyclonal anti-phospho-Akt [Ser473] (1:1000, Cell Signaling), rabbit monoclonal anti-Akt [pan] (C67E7, 1:1000, Cell Signaling), rabbit monoclonal anti-Bcl-2 (D55G8, 1:1000, Cell Signaling), rabbit polyclonal anti-Bax (2772, 1:1000, Cell Signaling), rabbit monoclonal anti-cleaved caspase-3 [Asp175] (5A1E, 1:1000, Cell Signaling) and rabbit monoclonal anti-cleaved poly (ADP-ribose) polymerase (PARP) [Asp214] (D64E10, 1:1000, Cell Signaling).

### siRNA-mediated knockdown of Sirt-1 and Sirt-3

Silencer select siRNA against human Sirt-1 and Sirt-3 along with the negative (non-specific) control-pool were purchased from Life Technologies Inc. Knockdown of Sirt-1 and Sirt-3 was performed similarly as previously described [12]. Briefly, cells were seeded into 6-well plates (300,000 cells/well) approximately 24 h before transfection in RPMI medium. On the day of transfection, 80 nM (40pmoles) Sirt-1, Sirt-3 or non-specific siRNA was first incubated with Lipofectamine 2000 (Invitrogen, Carlsbad, CA, USA) at room temperature for 30 min, then added to cells (~70% confluency) in Opti-MEM (low-serum) medium (Life Technologies Inc). After 4 h, the medium was aspirated and replaced with fresh RPMI medium. Twenty-four hours later the medium was aspirated and replaced with fresh RPMI medium ± 50 nM or 100 nM TL and cells cultured for an additional 20 h at 37°C. The knocked-down genes were confirmed by immunoblotting. All data were acquired from at least 3 independent experiments.

### SIRT-1/SIRT-3 enzyme inhibition by EX-527/nicotinamide

In brief, cells were seeded into 6-well plates (300,000 cells/well) approximately 24 h before treatment. Cells (~70% confluency) were then treated with either 10 μM EX-527 or 10 mM nicotinamide for 4 h at 37°C. Control cells were treated with medium containing 1× PBS or DMSO. After 4 h, the medium was aspirated and replaced with fresh RPMI medium ± 50 nM or 100 nM TL and cells cultured for an additional 20 h at 37°C. Inhibited gene expression was confirmed by immunoblotting. All data were acquired from at least 3 independent experiments.

### miR204-5p mRNA inhibition

MISSION miRNA inhibitor targeting human miR204-5p (Mature sequence: 5′-UUCCCUUUGUCAUCCUAUGCCU-3′) along with scrambled (control) miRNA were purchased from Sigma-Aldrich. Briefly, cells were seeded into 6-well plates (300,000 cells/well) approximately 24 h before transfection. On the day of transfection, 40 nM (20pmoles) of anti-miR204-5p or control miRNA was first incubated with Lipofectamine 2000 (Invitrogen) at room temperature for 30 min, then added to cells (~70% confluency) in Opti-MEM (low-serum) medium (Life Technologies Inc) at 37°C. After 6 h, the medium was aspirated and replaced with fresh RPMI medium. Twenty-four hours later medium was aspirated and replaced with fresh RPMI medium ± 50 nM or 100 nM TL and cells cultured for an additional 20 h at 37°C. All data were acquired from at least 3 independent experiments.

### Western blotting

Attached cells were collected by gentle scraping for protein extraction. Whole cell lysates were prepared by boiling cells for 10 min in a 2% SDS buffer (2% SDS, 50 mM Tris-HCl pH 6.8, 10% glycerol) supplemented with protease inhibitors (Sigma-Aldrich). Proteins were resolved on 8–10% SDS-PAGE followed by transfer to 0.2 μm nitrocellulose membranes (Bio-Rad Laboratories, Hercules, CA, USA). Western blotting was performed by incubating with appropriate primary antibodies followed by secondary antibodies conjugated to far-red fluorescent dyes (IRDye-680 and -800) and detection using the Odyssey LI-COR system (LI-COR Biotechnology, Lincoln, NE, USA). Blot quantifications were performed using LI-COR software. All data were acquired from at least 3 independent experiments.

### Real-Time RT-PCR

Total RNA and miRNA from cultured cells was isolated using miRNeasy Mini-Kit (Qiagen, Valencia, CA, USA) and cDNA was synthesized using qScript™ microRNA cDNA Synthesis Kit (VWR International, Radnor, PA, USA). Real-time PCR was performed using PowerUp SYBR Green Master Mix (ThermoFisher Scientific, Waltham, MA, USA) to monitor the amplification on an ABI-7500 PCR machine. The following primers were used: Human Caveolin-1 (Forward-CACATCTGGGCAGTTGTACC; Reverse-CACAGACGGTGTGGACGTAG), miR204-5p (Forward-CGCTTCCCTTTGTCATCCTA), Human Sirt-1 (Forward-TTGTTATTGGGTCTTCCCTCAAAG; Reverse-GACATCACAGTCTCCAAGAAGC), Human Sirt-3 (Forward-GAGCTTCTGGGCTGGACAGA; Reverse-TGGGATGTGGATGTCTCCTATG), Human SNORD44 (Forward-GCAAATGCTGACTGAACATGAA) and Human 18S (Forward-TAGAGGGACAAGTGGCGTTC; Reverse-CGCTGAGCCAGTCAGTGT). Real-time PCR assays were run in triplicate in MicroAmp Optical 384-well reaction plates on an ABI-7500 Real Time PCR System using the PowerUp SYBR Green Master Mix (ThermoFisher Scientific). RT-PCR reaction mixtures of a total volume of 10 μL contained 5 μL of 2× SYBR Green Master Mix, 0.5 μL each of 10 μM forward and reverse primers, and 2–3 μL cDNA. Cycling was performed using the default conditions of the Sequence Detection System software version 1.3 (Life Technologies Inc.): 10 min at 95°C, followed by 40 rounds of 15 sec at 95°C and 1 min at 60°C. Data were normalized to either the reference gene *18S* or *Snord44* (miR204-5p studies), a small non-coding RNA located within the nucleolus. All data were acquired from at least 3 independent experiments.

### Statistical methods and analyses

All data are representative of at least three independent experiments. Results are expressed as mean ± SD. For comparison to DMSO/non-TL-treatment, a one-sample *t*-test was conducted (μ = 1). For siRNA/enzyme inhibitor studies, differences were compared using two-sampled *t*-test. All analyses utilized α = 0.05, two sided. Analyses were conducted in R (v. 3.5.2).

## Abbreviations

Akt: RAC-alpha serine/threonine-protein kinase
CAV-1: Caveolin-1
EX-527: 6-Chloro-2,3,4,9-tetrahydro-1H-Carbazole-1-carboxamide
miRNA: MicroRNA
NAM: Nicotinamide
NSCLC: Nonsmall-cell lung cancer
PARP: Poly (ADP-ribose) polymerase
SIRT: Sirtuin
TL: Minnelide/Triptolide
